# Effects of cadmium on lipids of almond seedlings (*Prunus dulcis*)

**DOI:** 10.1186/s40529-014-0061-7

**Published:** 2014-08-02

**Authors:** Nada Elloumi, Mohamed Zouari, Leila Chaari, Chiraz Jomni, Brahim Marzouk, Ferjani Ben Abdallah

**Affiliations:** 1 Laboratory of Water, Energy and Environment, Sfax University, Higher Institute of Biotechnology of Sfax, Sfax, Tunisia; 2 Laboratory of Environment and Biology of Arid Area, Department of Life Sciences, Faculty of Sciences of Sfax, Sfax, Tunisia; 3 Laboratory of Water, Energy and Environment, Sfax University, ENIS, Sfax, Sfax Tunisia; 4 Laboratory of Substances Bioactives, Centre de Biotechnologie de Borj Cedria (CBBC), Hammam-Lif, 1050 Tunisia

**Keywords:** Cadmium, Galactolipids, Lipid composition, Neutral lipids, Phospholipids, Prunus dulcis

## Abstract

**Background:**

Cadmium uptake and distribution, as well as its effects on lipid composition was investigated in almond seedlings (*Prunus dulcis*) grown in culture solution supplied with two concentrations of Cd (50 and 150 μM).

**Results:**

The accumulation of Cd increased with external metal concentrations, and was considerably higher in roots than in leaves. Fourteen days after Cd treatment, the membrane lipids were extracted and separated on silica-gel thin layer chromatography (TLC). Fatty acid methyl esters were analyzed by FID-GC on a capillary column. Our results showed that Cd stress decreased the quantities of all lipids classes (phospholipids, galactolipids and neutral lipids). Galactolipid, phospholipid and neutral lipid concentrations decreased more in roots than in leaves by Cd-treatment. In almost all lipid classes the proportion of palmitic acid (16:0), linoleic (18: 2) and that of linolenic (18: 3) acid decreased, suggesting that heavy metal treatment induced an alteration in the fatty acid synthesis processes.

**Conclusions:**

In conclusion, our results show that the changes found in total fatty acids, in the quantities of all lipids classes, and in the in the profiles of individual polar lipids suggest that membrane structure and function might be altered by Cd stress.

**Electronic supplementary material:**

The online version of this article (doi:10.1186/s40529-014-0061-7) contains supplementary material, which is available to authorized users.

## Background

Contamination of the soil by heavy metals is a serious environmental problem with potentially harmful consequences to agriculture (Di Toppi and Gabbrielli [1999]; Robinson et al. [2001]; Certık et al. [2005]). In most plant species, heavy metals have been shown to affect a wide range of plant cellular activities, such as mineral distribution (Shukla et al. [2003]; Ben Ammar et al. [2005]; Elloumi et al. [2007]), sugar metabolism (Elloumi et al. [2007]), sulphate assimilation (Nussbaum et al. [1988]), photosynthetic processes (Krantev et al. [2008]; Velikova et al. [2011]), plant growth (Das et al. [1997]) and several enzyme activities (Schützendübel et al. [2001]; Sobkowiak et al. [2004]). Some of these effects are mediated through a general action on the membrane structure, resulting from an alteration in lipid composition. Hall ([2002]) showed that plant membrane structure may be considered as the first target for heavy metal toxicity. Although different plant species and varieties can be affected by high amounts of heavy metals, some species are tolerant to lower levels. To avoid heavy metals toxicity, the plants adopt various defense strategies including internal metal detoxification processes which may be achieved through both cellular and subcellular compartmentation or complexation with cellular ligands such as phytochelatins (Cobbett and Goldsbrough [2002]; Ma et al. [2005]). It appears that cell decompartmentalization and modification of membrane functions represent the first targets for metal toxicity (Meharg [1993]; Ouariti et al. [1997]; Nouairi et al. [2006a]). To evaluate this, we need to know to what extent metal can result changes in fatty acid composition of the major lipids of the cell membranes. Changes in the composition of fatty acids which affect the structural and functional properties of cell membranes are important in the acclimation of most types of plants (Martin et al. [1976]). Previous work showed that the ratio of unsaturated to saturated fatty acids play an important role in determining the physiological function of the plant tissue (Brenner [1984]; Certık et al. [2005]). Lipid molecules are essential building blocks for every membrane of a living cell. Membranes are sites for many specific enzymatic activities, transport ions and metabolites, and hormonal receptors. The composition of membrane lipids may be a factor in determining major biological properties of membranes that may influence biological changes of plants (Certık et al. [2005]).

There is little information in the literature about the effects of heavy metals on membrane lipid metabolism. Most of the studies have been conducted to evaluate lipid peroxidation (Chaoui et al. [1997]; Attila et al. [2004]; Elloumi et al. [2007]) and fatty acid composition in isolated chloroplasts of stressed plants (Vassilev et al. [2004]).

The aim of the present work was to assess how Cd stress affects the composition and biosynthesis of lipids and fatty acids in almond leaves and roots, accompanying the accumulation of this heavy metal in almond seedlings.

## Methods

### Plant culture

Seeds of *Prunus dulcis* were germinated on wet filter paper, in the darkness at 4°C for 15– 20 days. Following their germination, seedlings having approximately the same size were transferred to a Long Ashton nutrient solution (Hewitt [1966]) that was continuously aerated and weekly renewed. Seedlings were cultivated in a growth chamber under the following conditions: 22/26°C day/night temperature, 16/8 photoperiod and 300 μmol m^–2^ s^–1^ light intensity. After a 2-week acclimatization phase, 50 and 150 μM CdCl_2_ were added to the nutrient medium. On the 14th day from the start of Cd supply, eight plants from each treatment were harvested. Leaves, stems and roots were separated.

The roots seedlings were washed first with 20 mM Na_2_-EDTA for about 10 min in order to remove excess Cd adhering to the surfaces and finally washed with deionized water.

Dry matter of different organ tissues was determined after drying in an oven at 80°C for 48 h.

Dried samples were then ground and reduced to fine powder.

### Cadmium analysis

Ground samples were digested in a nitric and perchloric acid mixture (2:1 v/v). Cadmium was determined by atomic absorption spectrophotometry with polarized Zeeman (HITACHI; Z-6100).

### Lipid extraction and separation

Total lipids from leaves and roots were extracted using the method described by Bligh and Dyer ([1959]). One gramme of fresh material was fixed by boiling water for 5 min, in order to inactivate the phospholipases. Samples were ground manually in a mortar into a mixture of chloroform/methanol (2:1, v/v). After washing with water of fixation and decantation at 4°C, two phases were obtained. Then the organic phase containing total lipids was recovered and dried under a nitrogen stream. Finally, the residue was dissolved in a known volume of toluene–ethanol (4:1, v/v) and stored at -20°C for further analyses.

### Lipid analysis

PL, GL and neutral lipids (NL) were separated by TLC on silica gel plates 60 (Merck) with the following solvent mixtures: chloroform:acetone:.methanol:acetic acid:water (50:20:10:10:5, v/v) (Lepage [1967]). After development, bands were located with iodine vapours or spraying the plates with 0.1% Rhodamine 6G in ethanol. Individual lipids were identified by comparison with lipid standards and scraped from the TLC plates. The samples were quantified against a heptadecanoic acid (17:0) internal standard, and the amount of each lipid species in the sample could, therefore, be expressed both as an absolute amount in the 0.1 g samples, and as a percentage of the total lipid present in the sample.

Fatty acids from total lipids and lipid classes were transformed into their corresponding methyl esters as described by Cecchi et al. ([1985]). Transmethylation was made by the addition of 2 ml of hexane, 0.5 ml of 3% sodium methylate, a known amount of the internal standard heptadecanoic acid methyl ester (C17:0), 0.2 ml of 1 NH_2_SO_4_ and 1.5 ml of 10% sodium chloride. The hexanic phase containing fatty acid methyl esters (FAME) was recovered and its volume was reduced in a stream of nitrogen, prior to analysis.

GC analyses were performed using a Hewlett-Packard 6890 gas chromatograph equipped with a flame ionization detector (FID) and an electronic pressure control (EPC) injector. A polar HP Innowax (PEG) column (30 m × 0.25 mm, 0.25 μm film thickness) was used. The carrier gas was N_2_ with a flow rate of 1.6 ml/min. The split ratio was 60:1. The analyses were performed using the following temperature program: the initial oven temperature was held at 150°C for 1 min, increased at a rate of 15°C/min to 200°C, and then was held there for 3 min and finally ramped at a rate of 2°C/min to 242°C. The detector and injector temperatures were set at 275 and 250°C, respectively (Aidi Wannes et al. [2009]). Analyses were performed in triplicate. Fatty acid methyl esters were identified by comparison of their retention times to those of pure reference standards.

### Statistical analysis

Microsoft Excel and SPSS 17.0 were used to analyze the data. All measurements were replicated three times. Average values and standard deviations (S.D.) were calculated by Microsoft Office Excel 2007 for all the data in this paper. Duncan’s multiple range test was employed to compare the changes among different treatments at *p <* 0.05 level.

## Results

**Figure 1:**
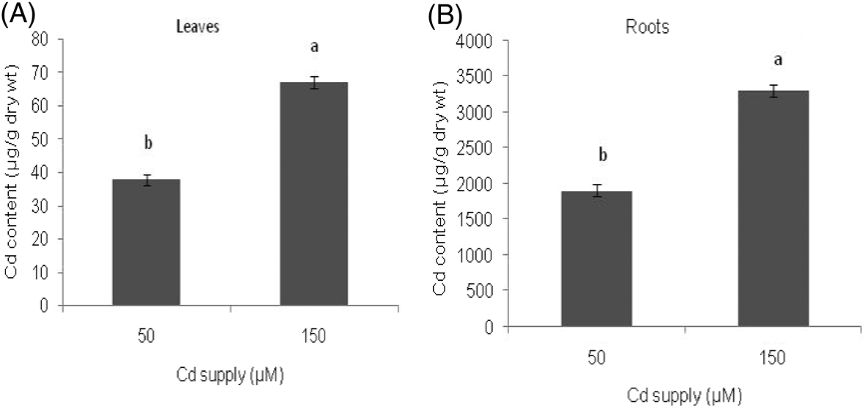
**Cadmium content in leaves (A) and roots (B) of almond seedlings after 14 days of exposure to two concentrations of cadmium.** The error bars indicate mean ± SE (n = 3). Means with different letters indicate a significant difference at P < 0.05 using Duncan multiple range test.

Almond plants exposed to Cd in the nutrient solution accumulated substantial amounts of Cd in the roots and the leaves (Figure 1). At 50 μM about 96% of total plant concentration of Cd was found in the roots. At 150 μM, this proportion increased to 98%, thus suggesting that the root is the primary site of Cd accumulation. Small amounts of Cd, however, were translocated into the leaves.

**Figure 2:**
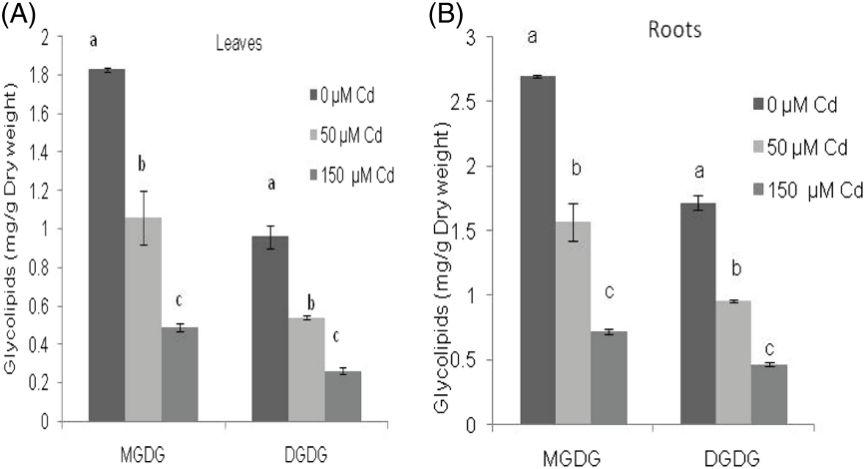
**Galactolipids classes of leaves (A) and roots (B) of almond seedlings exposed to two concentrations of cadmium.** The error bars indicate mean ± SE (n = 3). Means with different letters for each galactolipid class indicate a significant difference at P <0.05 using Duncan multiple range test.

**Figure 3:**
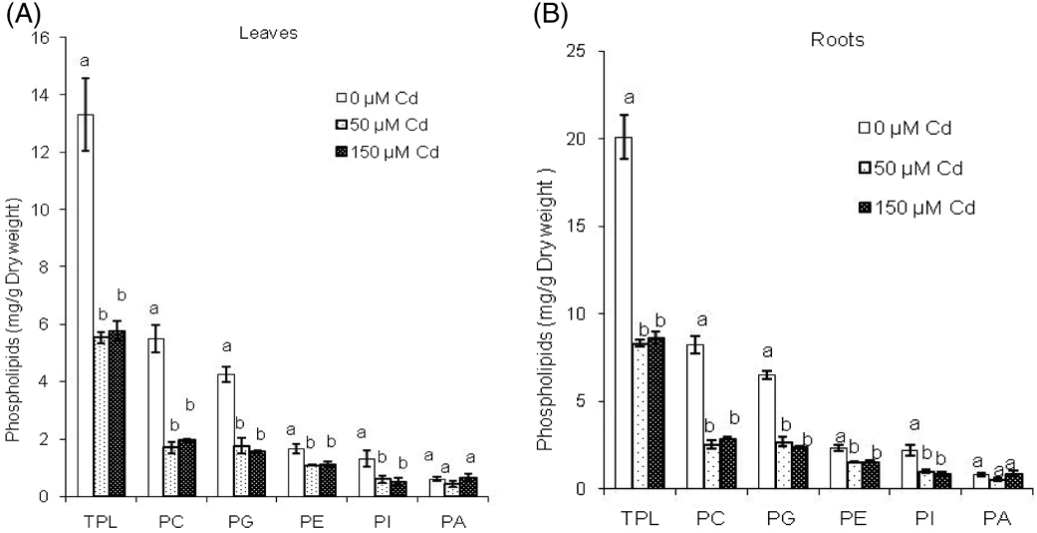
**Phospholipid classes of leaves (A) and roots (B) of almond seedlings exposed to two concentrations of cadmium.** The error bars indicate mean ± SE (n = 3). Means with different letters for each phospholipid class indicate a significant difference at P <0.05 using Duncan multiple range test. TPL, total phospholipids; PC, phosphatidylcholine; PG, phosphatidylglycerol; PE, phosphatidylethanolamine; PI, phosphatidylinositol; PA, phosphatidic acid.

In control plants, significant differences in the lipid composition of roots and leaves were observed (Table 1). In the leaves, galactolipids were the major constituent lipid classes, accounting for 51% of the total lipids (Table 1). Moreover, in this lipid class the proportion of monogalactosyl diacylglycerol (MGDG) was higher than that of digalactosyl diacylglycerol (DGDG) (29 and 21% of total lipids, respectively) (Figure 2). However, root lipids were characterized by a high level of phospholipids, about 64% of the total lipids, followed by neutral lipids and galactolipids (Table 1). The most abundant phospholipids in both leaf and root tissues were phosphatidylcholine (PC) and phosphatidylglycerol (PG) (Figure 3). It is also important to note that the percentage of DGDG of total root lipids was lower than that of MGDG (1.7 and 2.7% of total lipids, respectively). Some fatty acid species, palmitic (C16: 0), linoleic (C18:2) and linolenic (C18:3) acids were commonly higher in root and leaf lipids, and only differed in their relative proportions (Table 2). The contribution of C18:3 in the leaves was significantly high (48% of total fatty acids) and that of C16: 0 and C18:2 were low (14.6 and 13.3% of total fatty acids, respectively), and vice versa in the root lipids. Also palmitoleic (C16:l), stearic (C18:0) and oleic (C18:l) acids were present. Furthermore, the fatty acid composition of leaf lipids was characterized by the presence of C20: 0, approximately 9.7% of the total fatty acids (Table 2).

**Table 1:** **Lipid concentration (mg g-** ^**1**^ **dry wt) of leaves and roots of almond seedlings exposed to two concentrations of cadmium**

Treatments	Galactolipids	Phospholipids	Neutral lipids	Totals lipids
		Leaves		
Control	19.89 ± 3.22a	13.30 ± 1.26a	5.65 ± 0.55a	38.84 ± 2.22a
Cd 50	12.06 ± 0.84b	5.54 ± 0.19b	3.93 ± 0.37b	31.52 ± 0.30b
Cd 150	7.28 ± 1.02c	5.77 ± 0.36b	4.27 ± 0.23b	14.27 ± 1.13c
		Roots		
Control	2 .79 ± 0.045a	15.45 ± 0.096a	6.01 ± 0.003a	24.25 ± 3.89a
Cd 50	1.59 ± 0.154b	8.46 ± 0.37b	3.36 ± 0.058b	17.78 ± 2.86b
Cd 150	0.75 ± 0.0036c	4.59 ± 0.002c	2.10 ± 0.041c	6.26 ± 2.24c

Means with different letters indicate a significant difference at P <0.05 using Duncan multiple range test.

**Table 2:** **Total fatty acid composition of leaves and roots of almond seedlings exposed to two concentrations of cadmium**

	Treatments		
	C	Cd50	Cd150
Fatty acid of leaves (% of total)			
C16:0	14.6a	13.4a	13.9a
C16:1	1.6a	1.4a	1.9a
C18:0	2.1b	3.4a	3.6a
C18:1	10.3a	10a	9a
C18:2	13.3a	16.7a	14.3a
C18:3	48.4a	46.3a	48.3a
C20:0	9.7a	8.8a	9a
Fatty acid of roots (% of total)			
C16:0	23.4b	22.0b	27.1a
C18:0	7.1b	9.3a	8.3a
C18:1	10.3b	16.7a	8.3b
C18:2	40.0a	33.5b	32.4b
C18:3	19.2b	18.5b	23.9a

Means with different letters indicate a significant difference at P <0.05 using Duncan multiple range test.

The amount of total lipids, which corresponds to the total fatty acids, was lower in treated plants than in control ones, and was remarkably dependent on metal dose. The results (Tables 1 and 2) indicate a decrease of leaf lipids from 18% to 63% of the control with Cd treatment (Table 2). In roots, there was however a marked drop of total lipids and this ranged from 27 to 74% of the control with Cd treatments (Table 1). In leaves, the fatty acids most subject to variation were stearic (C18:0) acids (Table 2). However, in roots the remarkable impoverishment of 18: 2 was accompanied by enrichment of C16:0, C18:0 and C18:3.

The data in Table 1 reveal that the decline of total lipid concentration in treated seedlings was associated with a shift towards a low concentration of all lipid classes. Also, there were substantial differences in the effect of Cd on lipid classes isolated from leaves and roots of stressed plants. Indeed, the galactolipid concentration was more reduced in roots (74% of control) than in leaves (63% of control) by toxic Cd-treatment (Table 1). An important decrease of MGDG concentration (74% of control) and DGDG concentration (73% of control) was observed in metal stressed-almond leaves and roots. The MGDG/DGDG ratio remained, remarkably, unchanged in roots and in leaves (1.9) (Figure 2). Beside, the decrease in phospholipid concentration by Cd treatment was more pronounced in roots than in leaves; and this resulted in a decline of the concentration of all phospholipid molecules including phosphatidylcholine (PC), phosphatidylglycerol (PG), phosphatidylethanolamine (PE), phosphatidylinositol (PI) and phosphatidic acid (PA) (Figure 3).

The variation in neutral lipid content was also similar to that of the phospholipids. The changes in fatty acid composition due to heavy metal-stress differed from individual lipids as well as organs (Tables 3 and 4). Therefore, the fatty acids composition of galactolipids changed by Cd treatment. In fact, within MGDG of treated leaves the drop of C18:3 concentration results in an increase of C16:0 percentages (Table 4). Whereas, whithin the MGDG of treated roots the drop of C18:1and C18:2 concentrations results in an increase of C18:3 percentages. Further, within DGDG of treated roots the drop of C18:1concentration results in an increase of C18:3, C18:0, C16:1 and C16:0 percentages. In neutral lipids, the decrease of C20:0 and C18:1 percentages was accompanied with an augmentation of C18:3 and C18:2 in leaves. However, in roots the decrease of C18:1 percentage was accompanied with an augmentation of C18:3, C18:2 and C18:0. The fatty acid composition of phospholipids also changed by heavy metal-stress. Therefore, within both PC and PG of leaves the high Cd concentration resulted in a decrease of C18:1 and C18:3 proportions with an increase of C18:0. But in PG of stressed roots, the sharp decline of C16:0 and C18:2 percentages was coupled with an increase of C18:0 and C18:1 fatty acids. Moreover, within PC of treated roots we noted an increase only in that of saturated ones, particularly of C18:0 and C16:0. Although in phospholipid classes of leaves the changes of fatty acid composition were somewhat different from those of roots, our data generally indicate that the toxic level of the heavy metals used leads to a decrease in concentration of unsaturated fatty acids and to an increase in that of saturated ones.

**Table 3:** **Fatty acid composition of galactolipids, neutral lipids and phospholipids in roots of almond seedlings after exposure to two concentrations of cadmium**

Acyl		Fatty acid composition (% of total)						
Lipids	Treatment	C16:0	C16:1	C18:0	C18:1	C18:2	C18:3	Unsaturation
MGDG	0	22.8a	2.8b	8.5a	20.6a	31.6a	13. 7b	68.7
	50	21.5a	4.3a	5.4a	13.0b	27.2b	28.6a	73.1
	150	22.7a	6.3a	9.1a	11.7b	19.8c	30.4a	68.2
DGDG	0	26.4b	3.4b	11.5b	21.1a	31.6a	6.0b	62.1
	50	28.5b	3.9b	14.7a	13.8b	33.1a	6.0b	56.8
	150	31.1a	5.5a	13.9a	10.2b	27.3b	12.0a	55
PC	0	28.7b	1.7a	8.8b	9.1a	39.2a	12.4a	66.6
	50	33.6a	1.7a	7.9b	9.4a	33.6b	13.9a	47.6
	150	32.3a	1.1a	12.4a	7.1a	32.8b	14.3a	47.1
PG	0	67.3a	5.2a	7.6c	7.9c	9.2b	2.8a	53.6
	50	60.8b	5.6a	9.5b	10.8b	10.0a	3.3a	41.1
	150	59.4b	5.3a	13.7a	9.8a	8.7c	3.1a	49.0
LN	0	35.5a	7.6a	6.6c	22.4a	24.0b	3.9b	57.9
	50	28.2b	8.6a	9.2b	18.3b	28.4a	7.2a	62.5
	150	26.8b	9.3a	11.5a	19.4b	27.3a	5.7a	61.7

Means with different letters for each lipid class indicate a significant difference at P <0.05 using Duncan multiple range test.

**Table 4:** **Fatty acid composition of galactolipids, neutral lipids and phospholipids in leaves of almond seedlings after exposure to two concentrations of cadmium**

Acyl		Fatty acid composition (% of total)							
Lipids	Treatment	C16:0	C16:1	C18:0	C18:1	C18:2	C18:3	C20:0	Unsaturation
MGDG	0	4.5b		2.0a	7.5a	15.6a	70.4a		93.5
	50	7.3a		2.6a	9.9a	15.4a	64.8b		90.1
	150	6.8a		2.8a	8.9a	12.4a	69.1b		93.4
DGDG	0	31.3b		4.4a	3.1a	10.8a	50.4a		64.3
	50	30.1b		6.6a	2.7a	6.7b	53.9a		63.3
	150	40.1a		5.6a	2.8a	8.5b	43.0b		54.3
PC	0	25.6b		7.8b	35.1a	18b	13.5a		66.6
	50	37.7a		14.7a	21.2b	17.2b	9.2b		47.6
	150	38.9a		14a	16.6b	22.5a	8b		47.1
PG	0	44.1b	14.9b	2.3b	14.1a	17.2a	7.4a		53.6
	50	56.0a	19.9ab	2.9b	8.4b	8.5b	4.2b		41
	150	44.3b	23.1a	6.7a	8.6b	11.7b	5.6b		49
LN	0	27.9b	8.1a	2.8a	8.3a	6.3b	3.2b	43.4a	25.9
	50	30.9a	8.5a	3.2a	7.1a	5.5b	6.3a	38.5b	27.4
	150	29.9a	6.7a	3.7a	6.7a	9.9a	6.1a	37.0b	29.4

Means with different letters in each column indicate a significant difference at P <0.05 using Duncan multiple range test.

## Discussion

The distribution of Cd in the plant tissues (root and Leaf) indicated that 90% of the Cd was retained in roots and only a small proportion, was translocated to aerial parts. The retention of high amounts of Cd in the roots is a typical response of several plants (Verkleij and Schat [1990]; Mohamed et al. [2012]). This is an important factor, since the differential accumulation of Cd between leaves and roots might explain the lower damage of lipid metabolism in the above ground parts of stressed almond plants. On the other hand, the excess of absorbed metals in roots can be pumped largely into intracellular compartments, resulting to a high metal concentration in the biochemically active compartments. This confirms furthermore the marked damage recorded in the root systems.

Our results show a significant decrease of the amount of total lipids, which corresponds to total fatty acids, as Cd concentration of the treatment medium increases. This decrease is more pronounced in root lipids than in leaf. In leaves of plants treated with the high Cd dose, only the percentage of C18: 0 increased. However, in roots a remarkable impoverishment of C18:2 was accompanied with an enrichment of C18:3, C18:0, C16:0. Also, it should be noted that in roots, excess Cd had a strong effect on total lipid concentration and fatty acid composition. In previous studies (Verdoni et al. [2001]; Le Guédard et al. [2008]) carried out with lettuce and tomato seedlings in a controlled environment, we showed that the percentage of tri-unsaturated fatty acids in leaves decreased while the percentage of C18:2, and to a lesser extent C18:1 and C18:0, increased, when plants were grown for 14 d on metal-contaminated soils. Hence, we proposed that the leaf fatty acid composition, and more precisely the C18:3/(C18:0 + C18:1 + C18:2) ratio could be used as an indicator of metal bioavailability and of their adverse effects on plants. In accordance with these results, a decrease of the percentage of C18:3 and an increase of the percentage of the other 18-carbon fatty acids were also observed in leaves of *Brassica napus* and *Capsicum annum* seedlings grown in hydroponic solutions supplied with CdCl_2_ (Jemal et al. [2000]; Ben Youssef et al. [2005]). Similar results were observed in leaves of tomato grown in solutions containing copper or cadmium (Ouariti et al. [1997]; Djebali et al. [2005]).

Galactolipids and phospholipids are essential constituents to all biomembranes. Monogalactosyl diglyceride (MGDG) and digalactosyl diglyceride (DGDG) were the major galactolipids in chloroplast thylakoid (Williams et al. [1983]) and primarily occurred in almond leaves. The most abundant phospholipids in almond leaves and roots were phosphatidylcholine (PC) and phosphatidylglycerol (PG). Changes occurring in the lipid composition of these membranes will certainly modify their permeability, energy transduction capacity, and the activities of membrane-bound enzymes (Wang and Lin [2006]). Palmitic (C16:0), stearic (C18:0), oleic (C18:1), linoleic (C18:2), and linolenic (C18:3) acids occurred in galacto- and phospholipids in the almond leaf and root tissue membranes. Palmitic, linoleic, and a-linolenic were the predominant fatty acids in almond. In different tissues (leaves and roots), their proportions of fatty acids were different.

In fact, under cadmium treatment we noted a significant decrease of lipid concentration and marked alterations of fatty acid composition in all lipid classes. A same decrease of MGDG and DGDG was observed in cadmium stressed-almond leaves and roots, resulting in unaffected MGDG/DGDG ratio. The decrease in galactolipids observed in this study can be interpreted to show alterations of photosynthetic membranes. The decrease in MGDG and DGDG indicated that cadmium could affect the photosynthesis, since the structure of chloroplastic membranes could be affected. Many studies have reported that the ratio of MGDG/DGDG was adversely affected in plants growing under stressful conditions (Zhang et al. [1996]; Ouariti et al. [1997]; Wang and Lin [2006]; Nouairi et al. [2006b]). In various plant species, the increase of MGDG/DGDG ratio has been detected in response to heavy metals treatment (Zhang et al. [1996]; Chaffai et al. [2005]; Nouairi et al. [2006b]). Nouairi et al. ([2006b]) confirmed that this high MGDG/DGDG ratio can be correlated with a stress resistance of *B. juncea* in their observation. Skorzynska et al. ([1991]) showed that the decrease of MGDG/DGDG ratio, might be caused by a high galactolipase activity, which attacks preferentially MGDG. All those changes would mainly concern the photosynthetic apparatus, in which heavy metals, could disturb the architecture of the thylakoidal membranes (Maksymiec et al. [1992]; Ouzounidou et al. [1992]). Such disorganization in turn affects some light reaction processes, especially those associated with PS II activity (Sun et al. [2010]). The PSII complex contains a large number of lipid molecules (Jordan et al. [2001]). The biochemical analysis of the lipid content of PSII indicates the presence a number of lipids whose composition is similar to the lipid composition of the thylakoid membrane (Loll et al. [2007]). It is well known that in leaves, C18:3 fatty acid is mostly associated with galactolipids (MGDG and DGDG) which account for more than 85% of thylakoid lipids (Somerville and Browse [1991]) and that these two molecules are crucial for photosynthetic activities (Mizusawa and Wada [2012]). It was shown that seedlings with reduced amount of MGDG have disrupted photosynthetic membranes. This result shows a complete impairment of photosynthetic ability and the growth (Kobayashi et al. [2007]; Aronsson et al. [2008]). Similarly, DGDG deficiency results in disorganization in turn affects some light reaction processes, especially those associated with PS I activity that alter the flux of electrons through the photosynthetic electron chain and impair the regulation of distribution of excitation energy between the photosystems (Ivanov et al. [2006]). In fact, galactolipids and the tri-unsaturated fatty acid contents of leaves appear to be an important parameter in some aspects of plant physiology such as the efficiency of photosynthesis and the response to stress. It must be kept in mind that modifications in the leaf fatty acid composition may have a direct impact on the plant physiological responses and fitness. Our previous work showed a significant decrease in chlorophyll *a* and *b* as Cd concentration in the treatment medium increases (Elloumi et al. [2007]). Accordingly, the reduction in Chl *b* varied proportionally with Cd supply, the reduction in Chl *a* was significantly more affected by 100 and 150 μM Cd than by 25 and 50 μM Cd.

The two other types of lipids investigated here, phospholipids and neutral lipids, behave similarly to the galactolipids. These components are more affected in roots than in leaves of almond plants treated with Cd. This trend is detected in others species such as *Silene cucubalus* grown under toxic concentration of Cu (De Vos et al. [1993]) and tomato plants treated with either Cd or Cu (Ouariti et al. [1997]). In fact the decline in the polar lipid concentrations (galactolipids and phospholipids) could be associated with losses in intracellular membranes (Djebali et al. [2002]). These findings indicate a decrease in the size and number of chloroplasts. The decrease in the phospholipid levels of the root cell membranes will probably have profound effects on ATPase activity (Kennedy and Gonsalves [1989]) and permeability (De Vos and Schat [1991]). The degree of fatty acid unsaturation is important to maintain the membrane fluidity and to provide the appropriate environment for membrane functions (Xu and Beardall [1997]). Our data indicate that toxic level of Cd used leads to a decrease in concentration of unsaturated fatty acids and to an increase in that of saturated ones in phospholipid classes.

The quantitative and qualitative loss in lipids of treated almond plants suggests that Cd induces disturbance of the membrane lipid. Among these effects, the activity of lipoxygenase was enhanced (Clijsters et al. [1991]; Somashekaraiah et al. [1992]). This is responsible for catalysing lipid peroxidation by using unsaturated fatty acids as substrates. Furthermore, the products of the lipoxygenase reaction such as peroxy and hydroxyl radicals, are reactive and can result in further membrane lipid deterioration, and can also affect other macromolecules in the cells (De Vos and Schat [1991]; Stadman [1993]). Heavy metals are also involved in the production of activated oxygen species that actively induce peroxidation of membrane lipids (Halliwel and Gutteridge [1984]). In fact, it is possible to suppose that a decrease of enzymic free radical scavengers caused by heavy metal may also contribute to the shift in the balance of free radical metabolism towards accumulation, leading to breakdown of membrane lipids.

## Conclusions

Our results show that the changes found in total fatty acids, in the profiles of individual polar lipids, and in the unsaturation levels suggest that membrane structure and function might be altered by Cd stress. Consequently, Cd stress is presumed to affect membrane fluidity and permeability by producing an irreversible damage to cell functioning. It should be pointed out that these changes are likely to be attributed to toxicity effects of Cd on membrane lipids, rather than the defense responses. This study clearly showed that the decrease of membrane lipids in almond plants treated with Cd may be related to an enhanced rate of catabolism and/or to the suppression of lipid biosynthesis.

## Authors’ original submitted files for images

Below are the links to the authors’ original submitted files for images. Authors’ original file for figure 1 Authors’ original file for figure 2 Authors’ original file for figure 3
