# Supplementary figures and images for: Effects of cadmium on lipids of almond seedlings (Prunus dulcis)
Source: Bot Stud. 2014 Aug 2;55:61. doi: 10.1186/s40529-014-0061-7 (PMC5430368; doi:10.1186/s40529-014-0061-7)

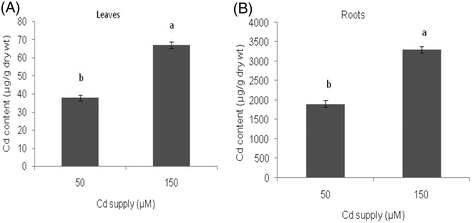

Supplement: Supplementary file 1 — Authors’ original file for figure 1 [file 40529_2014_9061_MOESM1_ESM.gif]

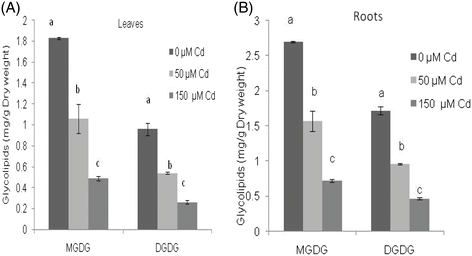

Supplement: Supplementary file 2 — Authors’ original file for figure 2 [file 40529_2014_9061_MOESM2_ESM.gif]

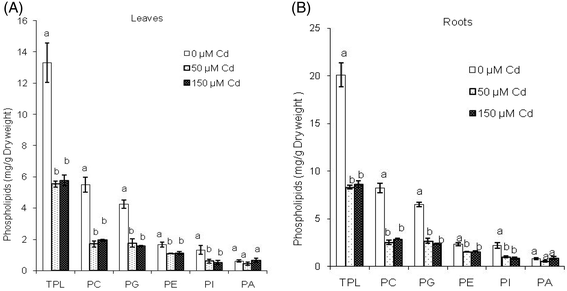

Supplement: Supplementary file 3 — Authors’ original file for figure 3 [file 40529_2014_9061_MOESM3_ESM.gif]
